# Effect of Moisture Condition of Brick–Concrete Recycled Coarse Aggregate on the Properties of Concrete

**DOI:** 10.3390/ma15207204

**Published:** 2022-10-15

**Authors:** Yonghua Wu, Zhaodong Qi, Mengdie Niu, Yuan Yao, Zuoqiu Luo, Kaifeng Zhang

**Affiliations:** 1 College of Materials Science and Engineering, Xi’an University of Architecture and Technology, Xi’an 710055, China; 2 China West Construction North Co., Ltd., Xi’an 701116, China

**Keywords:** brick–concrete recycled coarse aggregate, moisture condition of aggregate, compressive strength, autogenous shrinkage, pore structure

## Abstract

The application of brick–concrete recycled aggregates can alleviate the problem of increasing construction waste and increasing scarcity of natural aggregates. The different moisture condition of coarse aggregates can significantly affect the performance of brick–concrete recycled aggregate concrete. In this paper, the additional water quantity of dry and air-dried brick–concrete recycled coarse aggregate concrete was determined. Additionally, the fluidity, rheological parameters, autogenous shrinkage, strength and chloride ion penetration resistance were tested, and compared with saturated surface dry recycled brick–concrete coarse aggregate concrete and natural aggregate concrete. The results showed that the slump of concrete was increased, whereas the plastic viscosity, static and dynamic yield stress were decreased by adding additional water or using saturated surface dry coarse aggregate. Compared with the dry and saturated surface dry state, the air-dried recycled coarse aggregate concrete has the smallest 28 days autogenous shrinkage value, higher compressive strength and splitting tensile strength, and less adverse effects on chloride permeability. It is most beneficial to the performance and economy of concrete to adopt the air-dried state when the brick–concrete recycled coarse aggregate is applied in engineering.

## 1. Introduction

With the speedy development of construction projects within cities, there is a growing scarcity of natural aggregates that can be mined around cities. At the same time, in the process of urban construction, random accumulation of construction waste after the demolition of old buildings will cause environmental pollution and occupied arable land problems. In China, construction waste has accounted for 30~40% of urban waste, and the annual discharge of construction waste has reached more than 400 million tons [1,2]. Therefore, in recent years, the research on the preparation of recycled aggregates from construction waste has become increasingly extensive, and its application in engineering has been increasing. This is not only conducive to the recycling of construction waste resources, but also beneficial to the protection of natural sand and stone resources.

The recycled coarse aggregate has high water absorption due to its low density, high porosity, rough interface and mortar adhesion on the surface [3]. When the recycled coarse aggregate is directly mixed into concrete without pretreatment, the fluidity of the mixture will significantly decrease [4], and its strength, shrinkage performance and durability will be affected [5]. In China, many buildings in the last century are brick–concrete structures. After the demolition of these buildings, a large number of brick–concrete recycled aggregates containing broken brick particles will be produced [6]. Compared with the ordinary recycled aggregate, the brick–concrete recycled coarse aggregate has more pores and stronger water absorption [7,8,9]. In order to eliminate the adverse effects of excessive water absorption of recycled coarse aggregates, three processes are often used in the preparation of concrete with recycled coarse aggregates: (1) by using completely dry aggregates with sufficient additional water [10,11]; (2) by using saturated surface dry aggregates saturated by pre-absorbed water [12]; (3) by making surface modifications of aggregates to reduce their water absorption [13].

Ferreira [14] and Mefteh [15] confirmed that, when additional water was added to dry recycled aggregates, the initial fluidity of the concrete increased and the compressive strength of the formulated concrete was also higher than that when the pre-absorption process was used. Brand [16] also found that the initial slump of concrete prepared from dry state recycled aggregates was higher compared to natural aggregates for the same effective water binder ratio (W/B). However, when dry recycled aggregates are used, due to the huge difference in moisture content between the pastes and the aggregate, the high water absorption of the aggregate after mixing for a period of time causes rapid migration of water to the interior of the aggregate. This results in the effective W/B of the paste, especially interface transition zone (ITZ), is excessively reduced, which is not conducive to fluidity maintenance [17] and to interfacial bond strength [18].

The use of saturated surface dry recycled aggregate can reduce the water exchange between the aggregate and the cement paste [19], resulting in an increase in the initial fluidity of the prepared concrete [15]. However, Iris [20] showed that it was more difficult to control the fluidity of pre saturated recycled aggregate than that of dry aggregate. Moreover, Djerbi [21] concluded that compared with dry recycled aggregates, the interface zone of concrete prepared with saturated surface dry recycled aggregates has fewer pores. However, Poon [18] put forward that the use of saturated surface dry state of the recycled coarse aggregate, there was a “water release effect”, the water in the coarse aggregate will seep outward. Although it was beneficial to maintaining the slump of Concrete mixture, the W/B in the interface transition zone (ITZ) was increased. It will affect the interface bonding strength, and reduce the compressive strength. The same results were obtained in the experiments of Etxeberria [22] and Mefteh [15]. Oliveira [23] showed that compared with the dry state, the bending strength of the saturated recycled aggregate decreases more significantly. Li [11] pointed out that the higher the initial moisture condition of recycled fine aggregate, the greater dry shrinkage of mortar.

In order to reduce the water absorption of recycled aggregate, scholars have also investigated the modification of the surface of recycled aggregate using physical, chemical or microbial methods in recent years. He [24] used paste mixture of cement, silica fume and fly ash to impregnate the brick–concrete recycled aggregate, and then further treated with water glass, which could reduce the water absorption rate of the recycled aggregate by 42.4%. Kou [25] used polyvinyl alcohol (PVA) to impregnate the recycled aggregate, reducing the water absorption of the recycled aggregate from 6.23 to 1.62%. Zhang [26] treated the recycled aggregate with accelerated carbonation, which reduced the water absorption and the porosity within 10 μm of the recycled aggregate inner surface and. Wang [27] treated recycled aggregate samples with biological deposition also reduced the water absorption of the recycled aggregates, which increased the concrete compressive strength.

All of the above measures have certain effects on improving the properties of recycled aggregate concrete. However, baking the recycled aggregate to be complete dry, or pre-saturated by water absorption, as well as using different surface treatment processes, will substantially increase energy and manpower consumption, so that the cost of aggregates increased, which is not conducive to the large-scale application of recycled aggregates. The studies of Brand [16] and Iris [20] have pointed out that good mixing properties and mechanical properties can also be obtained by using air-dried recycled aggregate. However, there is little research on the air-dried recycled aggregate at present, and there is a lack of clear theoretical guidance on how to control the additional water volume of the air-dried recycled aggregate in engineering. Therefore, it is necessary to conduct a systematic study on the effect of air-dried recycled aggregate on the properties of concrete. In this paper, the influence of brick–concrete recycled coarse aggregate with three moisture conditions (dry, air-dried, saturated surface dry) on the performance of concrete is studied, so as to supply guidance for the application of brick–concrete recycled coarse aggregate.

## 2. Materials and Methods

### 2.1. Materials

Cement is the 42.5 R ordinary Portland cement of Jidong Heidelberg Cement Co, Ltd., (Xianyang, China), and the chemical composition of cement is listed in Table 1. The fly ash is class Ⅱ fly ash of Tongchuan Huaneng Power Plant, the physical properties of fly ash are displayed in Table 2. The polycarboxylic acid superplasticizer produced by China Construction Western Co., Ltd. (Xi’an, China), had a solid content of 14.4%. Fine aggregate is found in zone Ⅱ medium sand with a fineness modulus of 2.5. The brick–concrete recycled coarse aggregate (RA) is continuously graded from 5 mm to 20 mm, in which the content of crushed concrete particles is 65%, the content of broken brick particles is 34%, and the impurities are about 1%. The natural coarse aggregate (NA) used for comparison concrete is 5~20 mm Tongchuan limestone gravel, and the properties of coarse aggregate are listed in Table 3.

### 2.2. Test Methods

#### 2.2.1. Water Absorption Rate, Slump and Strength

The water absorption rate of coarse aggregate was carried out according to Chinese National Standard GB/T 14685-2011. First, soak the aggregate in water for 24 h, take out and dry the off-surface water with a wet towel, weigh the saturated surface dry mass, and then dry it in an oven at 105 °C. Now, weigh the dry mass and calculate the water absorption rate.

The slump of fresh concrete was tested with reference GB/T 50080-2016. Mixed concrete mixture in three layers into the slump cylinder (upper mouth diameter 100 mm, lower mouth diameter 200 mm, height 300 mm), each layer plug pounding 25 times, smoothing the surface and holding the slump cylinder up vertically, measuring the difference between the height of the cylinder and the highest point of the concrete after slumping is the slump.

The compressive strength and splitting tensile strength were measured based on GB/T 50081-2019; the size of the specimen is 100 mm × 100 mm × 100 mm, and the compressive strength is tested by standard curing until the specified age. The loading speed of compressive strength test and splitting tensile strength test are 0.5~0.8 MPa/s and 0.05~0.08 MPa/s, respectively.

#### 2.2.2. Rheological Parameters of Concrete

The ICAR rheometer (Gangyuan test instrument factory, Tianjin, China) was used to test the fresh concrete; the height and diameter of the blades used are 127 mm, and the inner diameter of the barrel is 286 mm. After the concrete is evenly mixed, put into the rheometer barrel, and the blades are made to rotate at a constant low loading rate (0.025 r/s) to obtain the torque versus time. The concrete presents linear elastic changes before the yield torque. After reaching the yield torque, the fresh concrete structure is destroyed, and flow begins to occur, and the torque value gradually decreases. The yield torque value corresponds to the yield stress that is the static yield stress *τ*_0,*S*_, see Equation (1).

(1) $$\tau_{0,S}=\frac{2T_{m}}{\pi D^{3}\left(\frac{H}{D}+\frac{1}{3}\right)}$$

where: *T_m_* is yield torque; *D* is leaf diameter; *H* is blade height.

The dynamic yield stress and plastic viscosity are obtained by measuring the flow curve of fresh concrete. Firstly, it is loaded at the highest speed of 0.5 r/s for 20 s to destroy the flocculation structure of concrete, and then divide it into 7 test points with equal decreasing speed at each test point, and each test point is loaded continuously at an interval of 5 s. The final end rate was 0.05 rad/s. Fit the torque–speed point obtained to obtain the relationship between torque and speed as Equation (2).

(2) T=Y+VN

where: *T* is torque, Nm; *Y* is related to *τ*_0_, Nm; *V* is related to μ, Nm·s; *N* is the rotational speed, rad/s.

Based on the Reiner–Riwlin Equation (3), the slope is linked to plastic viscosity, cylinder height and cylinder radius, while the intercept is related to dynamic yield stress, viscosity and cylinder radius. Therefore, the rheological parameters in Bingham model can be calculated.

(3) $$\Omega =\frac{T}{4\pi h\mu }\left[\frac{1}{R_{1}^{2}}-\frac{1}{R_{2}^{2}}\right]-\frac{\tau_{0,D}}{\mu }\ln\left[\frac{R_{2}}{R_{1}}\right]$$

where: Ω is speed, rad/s; *T* is torque, Nm; *H* is leaf height, m; *R*_1_ and *R*_2_ are blade radius and cylinder radius, respectively, m; *τ*_0,*D*_ is the dynamic yield stress, Pa; *μ* is the plastic viscosity, Pa·s.

#### 2.2.3. Autogenous Shrinkage and Resistance to Chloride Permeability

The autogenous shrinkage of concrete was tested via NELD-NES-type concrete shrinkage deformation measurement, in line with the non-contact method in GB/T 50082-2009, with specimen size of 100 mm × 100 mm × 515 mm, the test temperature of (20 ± 2) °C, relative humidity of (60 ± 50)%. On the basis of the electric flux method in GB/T 50082-2009, the chloride permeability resistance was tested by CABR-RCMP6 concrete chloride diffusion coefficient & electric flux tester.

#### 2.2.4. Micro Morphology and Pore Structure

After curing for 28 days, the concrete specimens were crushed, and small piece of concrete containing recycled coarse aggregate were taken and dried to constant weight after termination of hydration. After spraying gold treatment, the micromorphology of the interface area was analyzed by Quanta 650 FEG (FEI Company, Hillsboro, OR, USA) scanning electron microscope. Small pieces without coarse aggregate were dried to a constant weight and tested for pore structure using AutoPore V9600 mercury porosimeter (Micromeritics, Norcross, GA, USA).

## 3. Results and Discussion

### 3.1. Water Absorption Rate and Mix Proportion

**Figure 1:**
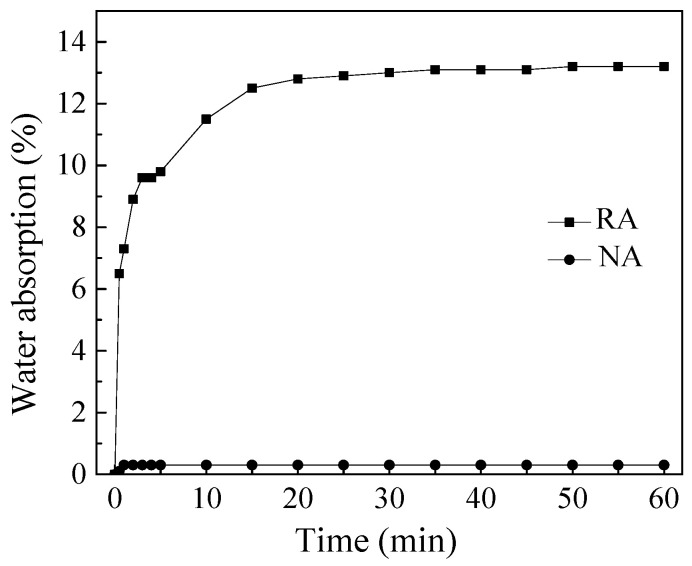
Time varying curve of water absorption for coarse aggregate.

Reasonable determination of additional water is the key to the application of recycled coarse aggregate in concrete. The quality moisture absorption rate of natural coarse aggregate and brick–concrete recycled coarse aggregate was measured separately with time, shown in Figure 1.

As shown in Figure 1, the saturated water absorption rate of NA was very small (0.3%), and it reached water absorption saturation in about 1 min; while RA contained a large amount of broken brick particles, the saturated water absorption was very high (14.1%), and its water absorption was a gradual process from fast to slow: the initial water absorption was fast, and the water absorption rate reached 6.5% at 1 min, and then continued to increase. It gradually stabilized after 20 min, and reached 13.2% at 60 min. Therefore, the water absorption characteristics of RA must be considered when preparing concrete. If dry RA was used, the additional water consumption was counted at the saturated water absorption of 14.1%, and the mixed concrete will occur serious bleeding phenomenon, which was caused by the inability of rapid absorption of excessive additional water. Through several experiments, the amount of additional mixing water was finally determined based on the 10 min water absorption of RA, and four kinds of concrete mix proportions were designed (see Table 4). In Table 4, the natural coarse aggregate was in a dry state, the porous recycled coarse aggregate was in a dry state, air-dried state and saturated surface-dry state, respectively. The four groups of concrete were recorded as NAC, RAC-G, RAC-Q and RAC-B, where NAC and RAC-B have no additional water, and for RAC-G and RAC-Q, the quantity of additional water was computed by the 10 min water absorption of RA.

### 3.2. Slump

**Figure 2:**
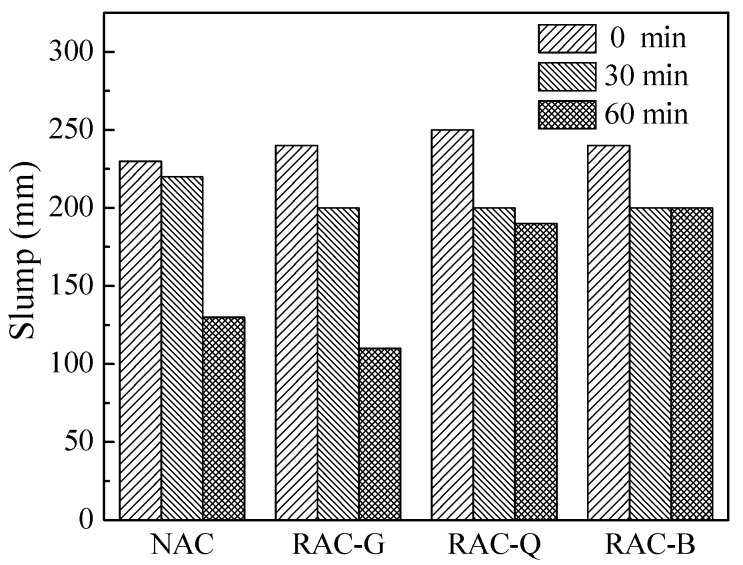
Slump of fresh concrete with different coarse aggregate.

The concrete was prepared according to the mix proportion in Table 4, and the slump of the mixture was measured, shown in Figure 2. The initial slump of RAC-G with additional mixing water, RAC-Q with additional mixing water and RAC-B with saturated surface dry were higher than that of NAC. This manifests that the initial fluidity of brick–concrete recycled coarse aggregate concrete can be increased by both pre-wetting and additional water processes. It is because the fluidity of concrete was intimately connected to the thickness of the water film on the surface of the particles composing the concrete [28]. As the increase of additional water and the gradual water-absorbing of brick–concrete recycled aggregate, the additional water cannot be completely absorbed in the initial stage, so the excess free water not adsorbed will increase the water film thickness on the particle surface, thus improving the slump of RAC-G and RAC-Q. On the contrary, In the RAC-B, the coarse aggregate has been saturated with water absorption in advance, so it will no longer absorb water from the paste. On the contrary, the water in the pores of the coarse aggregate also supplemented the free water in the paste on account of the “bleeding effect”, which increased the thickness of the water film and increased the slump.

It can also be seen from Figure 2 that the slump loss of NAC and RAC-G groups at 60 min is significant, and the slump at 60 min is 130 mm and 110 mm, respectively, which does not meet the requirement of GB/T 14902-2012 “Ready Mixed Concrete” that the slump value of pumped concrete is not less than 180 mm. While the slump loss of RAC-Q and RAC-B group is less. The slump loss of RAC-G, RAC-Q and RAC-B at 60 min were significantly different, which were 54.2%, 24.0% and 16.7%, respectively. The reason was that the additional mixing water initially added to RAC-G was largely absorbed by the dry coarse aggregate within 1 h, which significantly reduced the thickness of water film on the particles surface and thus caused an extremely reduction in the slump of concrete. In contrast, RAC-Q contained a certain amount of water in itself, the moisture gradient between the coarse aggregate and paste was less than that of dry coarse aggregate, and the water migration speed was slower, thus the slump loss at 60 min was smaller. As the brick–concrete recycled coarse aggregate was saturated with water, the slump loss of RAC-B was mainly caused by cement hydration and water evaporation, and the saturated brick–concrete recycled coarse aggregate also had a certain role of water supplement, so the slump loss at 60 min was minimal (see Figure 2).

### 3.3. Rheological Properties

**Figure 3:**
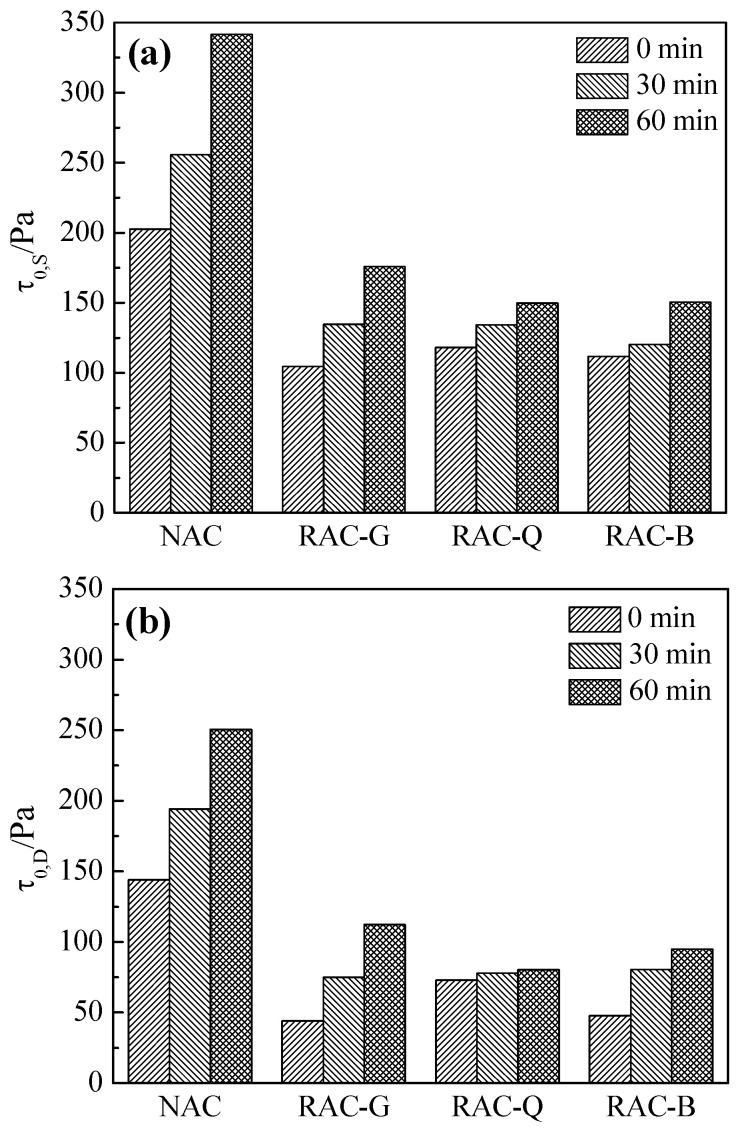
Effects of different coarse aggregates on the rheological properties of concrete (**a**) static yield stress; (**b**) dynamic yield stress.

Figure 3 shows the experimental results of static yield stress and dynamic yield stress of fresh concrete varying with time. The static yield stress is the minimum stress required to damage the fresh concrete flocculation structure at a smaller loading speed [29]; while the dynamic yield stress is gained from the rheological curve fitting, which represents the minimum shear stress required for the concrete to flow, and is related to the size of the adhesion and friction between particles [30].

From Figure 3a,b, the static yield stress and dynamic yield stress of NAC with natural aggregate are the highest at 0, 30 and 60 min, and the static and dynamic yield stress increase significantly with the prolonging of time. The static and dynamic yield stresses in the RAC-G, RAC-Q and RAC-B groups were lower due to the addition of additional mixing water or the pre-wetting of the aggregates. With the extension of time, the static yield stress and the dynamic yield stress of RAC-G also increased more, while the static yield stress and the dynamic yield stress of RAC-Q and RAC-B grew less, indicating that the super water absorption of dry aggregates made the W/B of cement paste decrease rapidly, and the flocculation structure of the paste is more easily enhanced, which is the same pattern as the slump test results. The dynamic yield stresses in Figure 3b are smaller than the corresponding static yield stresses in Figure 3a. This is because the dynamic yield stress is relevant to flow cessation, while the static yield stress is associated with flow initiation [31]. The difference between the two is a reflection of the thixotropy of the concrete mixture [32].

**Figure 4:**
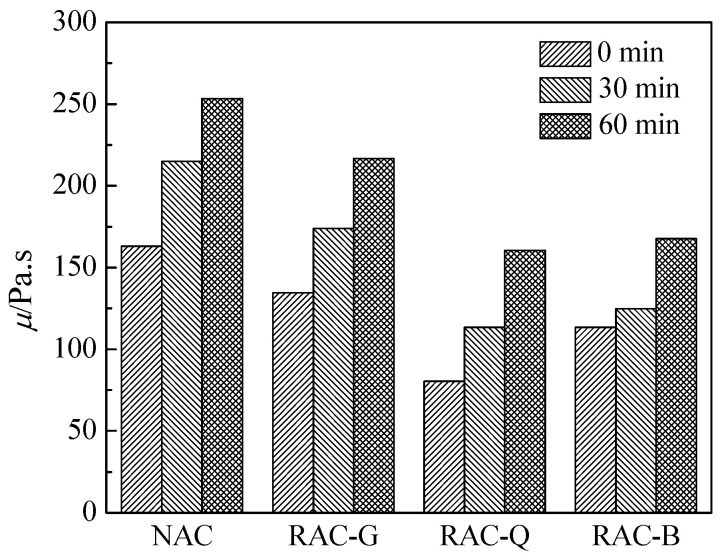
The effect of different coarse aggregates on the plastic viscosity of concrete.

The results of the variation of plastic viscosity of fresh concrete with time are shown in Figure 4. The plastic viscosity values of RAC-G, RAC-Q and RAC-B at 0, 30 and 60 min are smaller than those of NAC. The plastic viscosity reflects the rate of deformation of fresh concrete under the action of shear stress, which is mainly correlated with the amount of internal flocculation structures in the paste and the degree of damage [32], as well as the thickness of water film adsorbed on the particle surface [33]. Since additional water was added to RAC-G and RAC-Q, and RAC-B was pre-treated with water immersion, which increased the free water in the initial state and thickened the water film on the particle surface, so the plastic viscosity was lower than that of NAC group. With the progress of cement hydration process, the gravity between cement particles increases, the flocculation structure increases, the adsorbed water film becomes thinner, and the plastic viscosity increases. Compared with the RAC-G and RAC-B, the plastic viscosity of RAC-Q at 0 min, 30 min and 60 min was smaller, indicating that compared with the dry and saturated surface dry states, the moisture distribution inside the air-dried aggregate was more uniform and the adsorbed water film thickness was larger.

### 3.4. Autogenous Shrinkage

**Figure 5:**
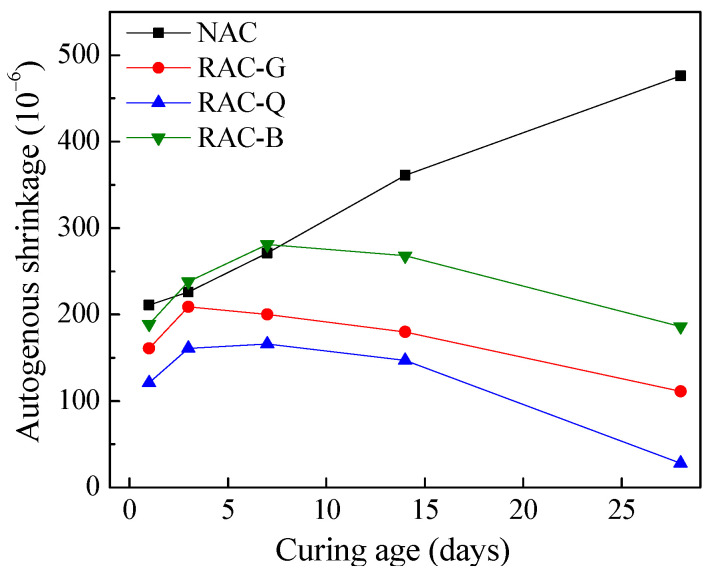
Autogenous shrinkage of concrete.

The autogenous shrinkage of concrete refers to the shrinkage attribute to the continuous hydration of cement under absolute humidity, which lead to a decrease in internal humidity and an increase in additional pressure generated by the curved liquid surface in the capillary tube. Due to the different moisture content of brick–concrete recycled coarse aggregate, the changes of internal humidity of concrete under absolute humidity were affected, and thus influence the autogenous shrinkage of the concrete. Four groups of concrete autogenous shrinkage were measured, the test results are represented in Figure 5.

From Figure 5, the order of 1 d autogenous shrinkage from largest to smallest was NAC > RAC-B > RAC-G > RAC-Q. This is owing to the autogenous shrinkage value was related to the W/B, and generally the smaller the W/B of cement paste, the larger the autogenous shrinkage [34]. NAC and RAC-B did not add additional water, but RAC-B had “bleeding effect”, and its actual W/B of cement paste was larger than that of NAC. Both RAC-G and RAC-Q added additional water, and the actual W/B of paste was larger than that of NAC and RAC-B. However, RAC-G had a great humidity difference between the dry coarse aggregate and the paste, and its water absorption rate was fast and water absorption capacity was large, resulting in a rapid decline in the actual W/B of the initial paste. While RAC-Q used air-dried coarse aggregate with a certain moisture inside, the humidity difference between the coarse aggregate and paste was little, and the water absorption rate was slow, which makes the actual W/B of the paste larger than that of RAC-G. Therefore, the order of the actual W/B of the cement paste from small to large was NAC < RAC-B < RAC-G < RAC-Q, which coincides with the rule of 1 d autogenous shrinkage. The research of Brand [16] also showed that the early autogenous shrinkage of brick–concrete recycled aggregate concrete was smaller than that of ordinary aggregate concrete.

As seen in Figure 5 that the autogenous shrinkage value of NAC was sustainably increasing within 28 days. This was because of the continuous hydration of cement, which caused a constant decrease in free water and humidity in the concrete, resulting in a continuous increase in autogenous shrinkage. RAC-G, RAC-Q and RAC-B prepared with recycled brick–concrete coarse aggregate obviously inhibited the autogenous shrinkage of concrete after 3~7 days, which significantly reduced the autogenous shrinkage of concrete. The reason is that the recycled coarse aggregate of brick–concrete in RAC-B contained water itself, while the recycled coarse aggregate of brick–concrete in RAC-G and RAC-Q absorbed a lot of water from the paste in the initial period. Therefore, when the internal moisture of concrete decreased in the later period, it is able to release water continuously to maintain sufficient humidity inside the concrete and act as an internal curing, so that the autogenous shrinkage was significantly reduced.

**Figure 6:**
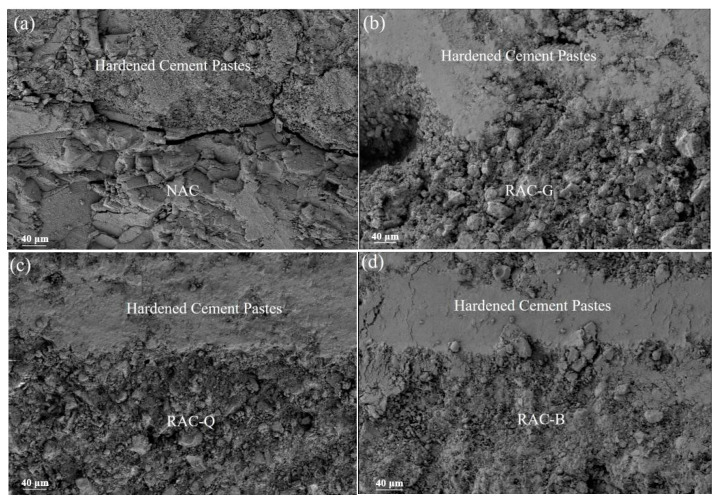
Micro morphology of interface between different coarse aggregate and paste.

The influence of brick–concrete recycled coarse aggregate on autogenous shrinkage can also be confirmed by the micro structure of the interface transition zone (ITZ) between coarse aggregate and cement stone. Figure 6 show the SEM pictures of the interface between different coarse aggregate and paste at 28 days.

From Figure 6a, there are obvious gaps at the interface between the natural coarse aggregate and pastes in the NAC. This is owing to the ordinary coarse aggregate concrete cement pastes were constantly hydrated, and the moisture in the capillary porosity continued to induce, making the curvature radius of the capillary concave liquid surface continuously decreased. And it is known from Laplace equation that the smaller the curvature radius of the concave liquid surface, the greater its additional pressure, thus increasing the additional pressure of the capillary pores, and then generating tensile stress in the cement stone. When the shrinkage stress in the ITZ between coarse aggregate and paste exceeded the limit value of interface bond strength, it cased cracks in ITZ. In Figure 6b–d, the interfaces between brick–concrete recycled coarse aggregate and cement paste in RAC-G, RAC-G and RAC-B were all tightly bonded without cracks, and the similar results were obtained in the study of Djerbi [21]. The reason for this that when the water in the capillary pores decreased because of the hydration of cement, the brick–concrete recycled coarse aggregate was able to constantly release the moisture stored in the pores and avoid the reduction of the curvature radius of the concave liquid surface in the capillary pores [11], which also avoid causing significant shrinkage and cracking.

By comparing Figure 6b–d, it can be found out that there are some differences in the hydration degree of cement at the interface of brick–concrete recycled coarse aggregate. In Figure 6b, the hydration degree of cement near the coarse aggregate was the worst in RAC-G, and there were many loose and pores on both the coarse aggregate side and the cement stone side. This may be caused by the rapid and large water absorption of the dry coarse aggregate, resulting in a low W/B of ITZ, which prevents the cement from being fully hydrate. Moreover, due to the rapid reduction of the W/B at the interface, the dissolution rate of cement particles was slow and the dissolution amount was small, and the cement hydration products cannot migrate to the inside of the recycled coarse aggregate, thus there were many pores on side of the coarse aggregate and the structure was loose. In Figure 6c, the cement stone at the interface of RAC-Q was dense without obvious pores, indicating that the cement hydration at the interface was more adequate. Compared with RAC-G, the coarse aggregate side of RAC-Q was also denser. The reason may be that the air-dried coarse aggregate contained certain water, and the humidity gradient between the interior of the coarse aggregate and the cement paste was lower, so the water absorption was tardy. The cement hydration in the interface area was relatively sufficient, and a certain amount of cement hydration products dissolved and migrated to the pores on the recycled aggregate surface, which made up for the defects on the recycled coarse aggregate surface and improved the ITZ structure significantly. In Figure 6d, the cement stone at the interface of RAC-B was the densest, and there was no obvious loose and pore space on the side of coarse aggregate, which means that the cement hydration at the interface of saturated coarse aggregate was the most adequate. This is because there was the “water release effect” in the saturated coarse aggregate, and the W/B of ITZ was relatively large. The ions generated by cement hydration can migrate farther, and more hydration products can be formed in the pores on the coarse aggregate surface, thus making the structure of the interface area more compact. However, it should be pointed out that although the apparent morphology at the interface of RAC-B was denser, some studies have shown that the hardness and elastic modulus of ITZ of RAC-B were lower due to the relatively higher W/B of ITZ [16], and this is detrimental to the strength growth.

### 3.5. Compressive Strength and Splitting Tensile Strength

**Figure 7:**
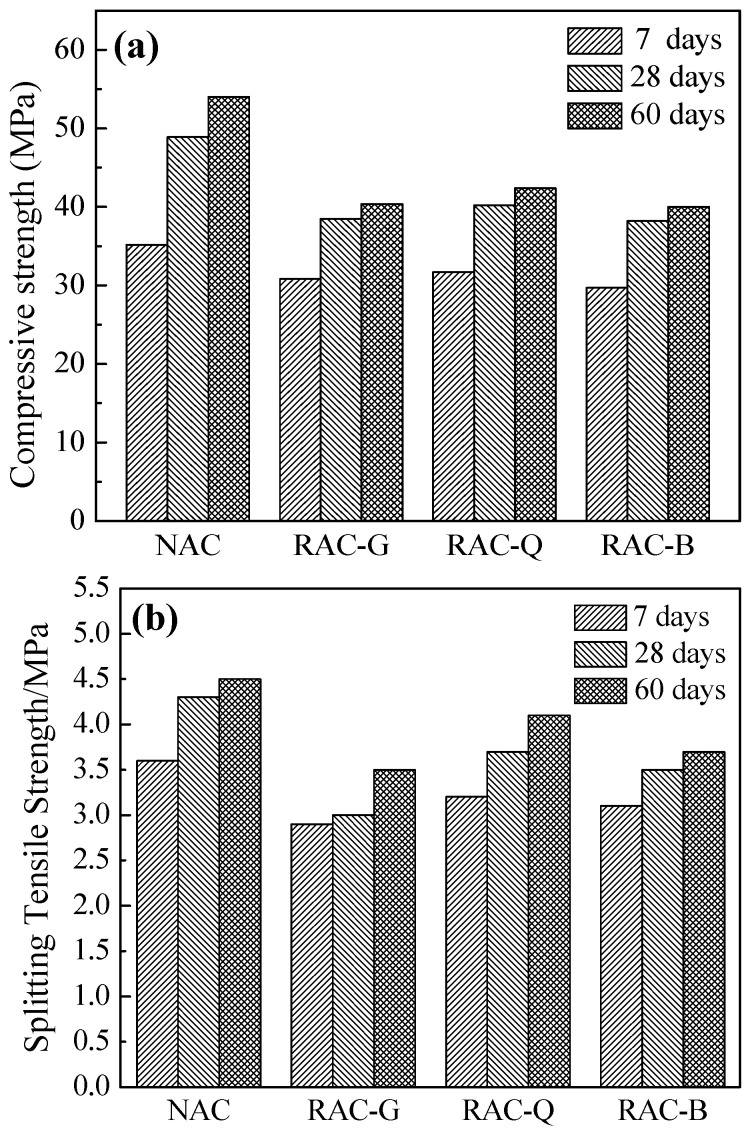
Compressive strength of concrete (**a**) and splitting tensile strength of concrete (**b**).

The concrete was prepared on the basis of the proportion in Table 3 and tested for compressive and splitting tensile strength. The results are displayed in Figure 7.

In Figure 7a, since the coarse aggregate of RAC-G, RAC-Q and RAC-B was recycled coarse aggregate and contained certain crushed brick particles, and the crushing index value was larger, thus the compressive strength of RAC-G, RAC-Q and RAC-B was smaller than that of NAC. In the three groups of brick–concrete recycled coarse aggregate concrete, the compressive strength of RAC-G and RAC-B at each age was lower than that of RAC-Q, indicating that the water saturated and dry state were more detrimental to the compressive strength of concrete compared to the air-dried state. The results of the 7 days, 28 days and 60 days splitting tensile tests in Figure 7b are similar to the compressive strength law, which also shows that the incorporation of all three water-content states of recycled aggregates reduced the splitting tensile strength of the concrete, with the air-dry state of the RAC-Q group having the least adverse effect on the 28 days and 60 days splitting tensile strengths. The reason that the additional water quantity of RAC-G was extremely greater than that of RAC-Q, which increased the effective W/B. Furthermore, the early rapid water absorption of recycled aggregate caused low W/B in the interface zone, which made the ITZ structure loose and porous (see Figure 6b) and lead to a reduction in strength. Owing to the “water release effect” of the saturated coarse aggregate in RAC-B, the W/B of ITZ was increased, and the hardness and elastic modulus of ITZ was reduced, thus affecting the strength of concrete.

Although the crushing index of brick–concrete recycled coarse aggregate was larger, it was mainly caused by crushed brick particles. Most of brick–concrete recycled coarse aggregate is still broken concrete particles (which also contains some natural coarse aggregate with mortar stuck to it), and crushed brick particles only account for a little portion. Therefore, the compressive strength at 28 days and 60 days can reach about 40 MPa [10], which indicates that the brick–concrete recycled coarse aggregate can be used for the preparation of medium and high strength concrete.

### 3.6. Chloride Ion Permeability Resistance

**Figure 8:**
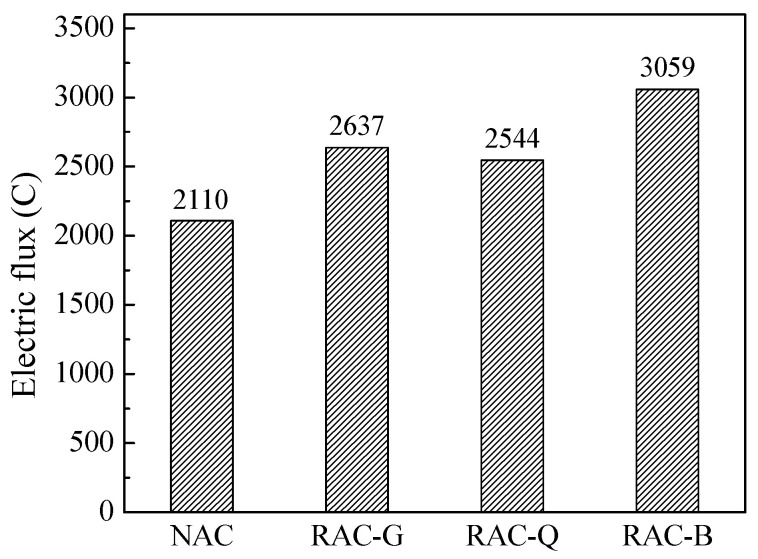
Electric flux of concrete.

Resistance to chloride permeability of brick–concrete recycled coarse aggregate concrete with different moisture conditions was tested using the electric flux method, as shown in Figure 8.

**Figure 9:**
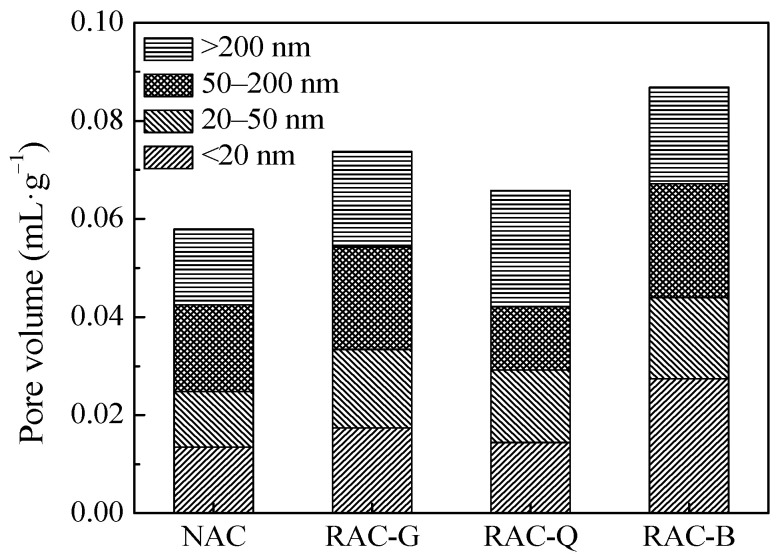
The volume of different pores in the mortar in the concrete.

As exhibited in Figure 8, the 28 days electric flux values of RAC-G, RAC-Q and RAC-B were greater than those of NAC, but they were all between 2000~4000 C, which were judged to be moderate chloride permeability in the light of ASTM C 1202-05 standard. The electrical flux of brick–concrete recycled coarse aggregate concrete with different moisture content state was in the order of RAC-B > RAC-G > RAC-Q, indicating that the dry state and saturated surface dry state were more disadvantageous to the chloride ion permeability resistance compared with brick–concrete recycled coarse aggregate in air-dried state. Porosity is an important factor affecting chloride permeability. The pore volume distribution of different pore diameters in the mortar part without coarse aggregate in the 28 days hardened concrete was tested, as displayed in Figure 9.

In Figure 9, the total pore volume of the mortar parts of RAC-G, RAC-Q and RAC-B was greater than those of NAC, which indicates that the pore structure of the mortar part was deteriorated by either the additional water or the water saturation process. The reason may be that the water absorption (dry and air-dried state) and water release (saturated surface dry state) effects of the recycled coarse aggregate, which caused the water transfer in the cement mortar and caused the increase of the pores of the cement mortar. In brick–concrete recycled coarse aggregate concrete, the pore volume of RAC-B was the highest. There exists water release effect in RAC-B, which increased the W/B of cement paste. Another aspect, although additional water was added to RAC-G and RAC-Q, the quantity of additional water added was computed based on water absorption rate in 10 min, which was less than the saturated water absorption. The additional water will be absorbed by the brick–concrete recycled coarse aggregate, and the actual effective W/B of the cement paste was smaller than that of RAC-B, as a result the pore volume of the mortar part of RAC-B was the highest. The reason for the pore volume of RAC-G mortar was higher than that of RAC-Q may be that the additional water volume in RAC-G was greater than that of RAC-Q, and the humidity gradient between RAC-G paste and coarse aggregate was greater than that of RAC-Q. Therefore, during the process of water migration, the osmotic pressure was greater and the damage to the structure was more significant, leading to the increase of the pore volume in RAC-G.

It should be illustrated that the pores in the mortar part were different from those in the interface zone of Figure 6. The pores in the interface zone were the structure of the interface micro area, which were mainly affected by the W/B of ITZ and the hydration degree of cement. While the pores in the mortar part reflected the sum of gel pores, capillary pores and coarse pores inside the cement stone, which were mainly affected by the effective W/B and water transfer (water absorption and bleeding).

The durability of concrete is mainly influenced by harmful pores and multi-harmful pores with pore diameter >50 nm [35]. According to the test results in Figure 9, the pore volumes of harmful pores and multi-harmful pores with pore diameter >50 nm in NAC, RAC-G, RAC-Q and RAC-B samples were 0.033 mL/g, 0.040 mL/g, 0.037 mL/g and 0.043 mL/g, respectively, that is, RAC-B > RAC-G > RAC-Q > NAC, which was consistent with the law of electrical flux test.

### 3.7. Discussion

From the previous test results, it can be seen that the concrete formulated with brick–concrete recycled coarse aggregate in the air-dried state is better than the recycled coarse aggregate in the dry and saturated surface-dry state in terms of fluidity, rheological behavior, and strength and durability. At present, many researches still focus on the use of saturated surface drying process [15], which was often used in lightweight aggregate concrete in the past [36]. Compared with brick–concrete recycled aggregates, the ordinary recycled aggregates (with waste concrete crushed particles mainly) have less surface porosity and lower water absorption [3], so this method can be used for reference. However, the pore structure of brick–concrete recycled aggregates is very different from that of lightweight aggregates: although the total porosity of lightweight aggregates such as clay ceramsite and fly ash ceramsite is higher (up to 50% or more), most of them are closed pores with less open pores (generally <10%) [37]. The pores of brick slag particles in brick–concrete aggregate are almost all open pores, and the open porosity can reach 20~30%. In this way, when the lightweight aggregate pre-wetting, only the surface open pores absorb water, and water absorption is limited, so there will be no serious “bleeding effect” when the concrete is mixed. However, brick–concrete recycled aggregate contains a large amount of water in the pores after pre-wetting and saturation, which is very easy to occur “bleeding effect”, affecting the mechanical property and durability of concrete.

On the other hand, due to the high water absorption of the brick–concrete coarse aggregate, the water absorption effect of the dry aggregate is also very significant, which leads to the obvious deterioration of the fluidity of the concrete mixture over time. Moreover, owing to the excessive competition for water from the paste by aggregate, the ITZ structure between the aggregate and the cement paste becomes worse, which affect the strength and durability. Therefore, it is better to use the air-dried state than the dry and saturated surface dry state for the brick–concrete recycled coarse aggregate.

However, it is important to noted that the additional water consumption in the concrete mix proportion should be calculated based on the water absorption rate of about 10 min of immersion, rather than the water adsorption saturation state of 24 h when using the air-dried state recycle aggregate. Otherwise, the concrete mix will be segregated, water secretion phenomenon, and affect the mechanical properties and durability. The research in this paper can provide guidance for the application of brick–concrete recycled coarse aggregate in engineering.

## 4. Conclusions

In this paper, according to the law of water absorption over time, the mixture ratio of recycled coarse aggregate concrete in dry, air-dried and saturated surface dry state was designed, and the performance of concrete was tested.

(1) Compared with the natural aggregate concrete, the initial slump of concrete was promoted by using dry and air-dried brick–concrete coarse aggregate and add extra mixing water, or using saturated surface dry coarse aggregate, but the slump loss of concrete was greater when dry brick–concrete coarse aggregate was used.
(2) Compared with dry and saturated surface dry states, the static and dynamic yield stress of the air-dried brick–concrete recycled coarse aggregate concrete increased the least with 60 min and changed steadily. At the same time, the plastic viscosity at 0, 30 and 60 min of the recycled coarse aggregate in air-dried state is the minimum.
(3) The autogenous shrinkage was reduced and the structure of ITZ was improved by using saturated or additional water technology for brick–concrete recycled coarse aggregates. Compared with the dry and saturated surface dry state, the autogenous shrinkage of the air-dried brick–concrete recycled coarse aggregate concrete is the smallest within 28 days.
(4) The compressive strength and splitting tensile strength of concrete prepared with brick–concrete recycled coarse aggregate concrete were reduced compared to the natural aggregate concrete. In comparison with dry and saturated surface dry states, brick–concrete recycled coarse aggregate in air-dried states has less adverse impact on the compressive strength and splitting tensile strength of concrete at 7, 28 and 60 days.
(5) Compared with the dry state and the saturated surface dry state, the amount of harmful pores and multi-harmful pores of concrete prepared by brick–concrete recycled coarse aggregate in air-dried state was the lowest, the 28 days electric flux value was the smallest, and the resistance to chloride ion permeability was best.

In summary, compared with the dry state and the saturated surface dry state, the performance of the air-dried brick–concrete recycled coarse aggregate is better. However, the increase is not significant, considering that the drying or water-saturated treatment of the coarse aggregate needs to increase a mass of energy and artificial costs. Therefore, it has the best comprehensive technical and economic benefits to directly use the air-dried brick–concrete recycled coarse aggregate and add extra water reasonably in the project.

## Figures and Tables

**Table 1:** Chemical composition of cement (%).

SiO_2_	Al_2_O_3_	Fe_2_O_3_	CaO	TiO_2_	MgO	SO_3_	Na_2_O	K_2_O
22.1	4.32	2.53	65.9	0.14	1.54	2.32	0.12	0.54

**Table 2:** Physical properties of fly ash.

Fineness (40 μm Sieve Residue) (%)	Water Demand Ratio (%)	Specific Surface Area (m^2^/kg)	Activity Index (%)
11.2	105	430	80%

**Table 3:** Physical properties of coarse aggregates.

Type	Apparent Density (kg/m^3^)	Bulk Density (kg/m^3^)	Porosity (%)	Mass Water Absorption (%)	Crushing Index (%)
NA	2750	1497	45.6	0.3	9.6
RA	1950	1100	43.6	14.1	20.0

**Table 4:** Mix proportion of concrete.

Number	Material Dosage (kg/m^3^)							Superplasticizer (%)	Moisture Content of Coarse Aggregate (%)	Apparent Density (kg/m^3^)
	Cement	Water	Additional Water	Fly Ash	Sand	NA	RA			
NAC	346	156	0	148	883	886	—	2.0	0	2419
RAC-G	346	156	72.2	148	883	—	628.3	2.0	0	2234
RAC-Q	346	156	46.5	148	883	—	654.0	2.0	4.1	2234
RAC-B	346	156	0	148	883	—	716.8	2.0	14.1	2250

## Data Availability

The general data are included in the article. Additional data are available on request.
